# Acute airway compromise due to parathyroid tumour apoplexy: an exceptionally rare and potentially life-threatening presentation

**DOI:** 10.1186/s12902-017-0186-2

**Published:** 2017-06-21

**Authors:** Aoife Garrahy, David Hogan, James Paul O’Neill, Amar Agha

**Affiliations:** 1 Department of Endocrinology, Beaumont Hospital, Dublin, Ireland; 2 Department of Otolaryngology, Head and Neck Surgery, Beaumont Hospital, Dublin, Ireland

**Keywords:** Parathyroid haemorrhage, Parathyroid tumour apoplexy, Primary hyperparathyroidism

## Abstract

**Background:**

Spontaneous haemorrhage into a parathyroid adenoma is a rare and potentially life-threatening presentation.

**Case presentation:**

We report the case of a 45 year old female recently diagnosed with primary hyperparathyroidism who presented with chest discomfort and acute airway compromise due to spontaneous extracapsular haemorrhage into a parathyroid adenoma. Computed tomography (CT) imaging showed a hypopharyngeal haematoma extending 10 cm into the superior mediastinum. Surgical decompression of the cyst followed by enbloc resection of the parathyroid tumour was performed after elective intubation. Calcium and parathyroid hormone (PTH) levels had fallen prior to surgery and remain normal post-operatively.

**Conclusion:**

Spontaneous parathyroid haemorrhage should be considered in any patient with unexplained spontaneous cervical haemorrhage, particularly if there is a history of hyperparathyroidism. Initial evaluation of such patients should include serum calcium and PTH as well as imaging.

## Background

Spontaneous parathyroid haemorrhage is an exceptionally rare but potentially life-threatening presentation. Haemorrhage may be contained within the gland, but often presents as extracapsular haemorrhage extending into the neck or mediastinum, manifesting as acute painful neck swelling, discomfort and cervical ecchymosis. Tracheal compression may lead to airway compromise while compression of the recurrent laryngeal nerve and oesophagus may lead to hoarseness and dysphagia respectively. We describe a case of spontaneous haemorrhage into a parathyroid adenoma presenting with acute airway compromise requiring surgical evacuation.

## Case Presentation

A 45 year old female presented to the emergency department (ED) with a 3 days history of progressive dyspnoea, cough, throat and chest discomfort and palpitations.

**Fig. 1:**
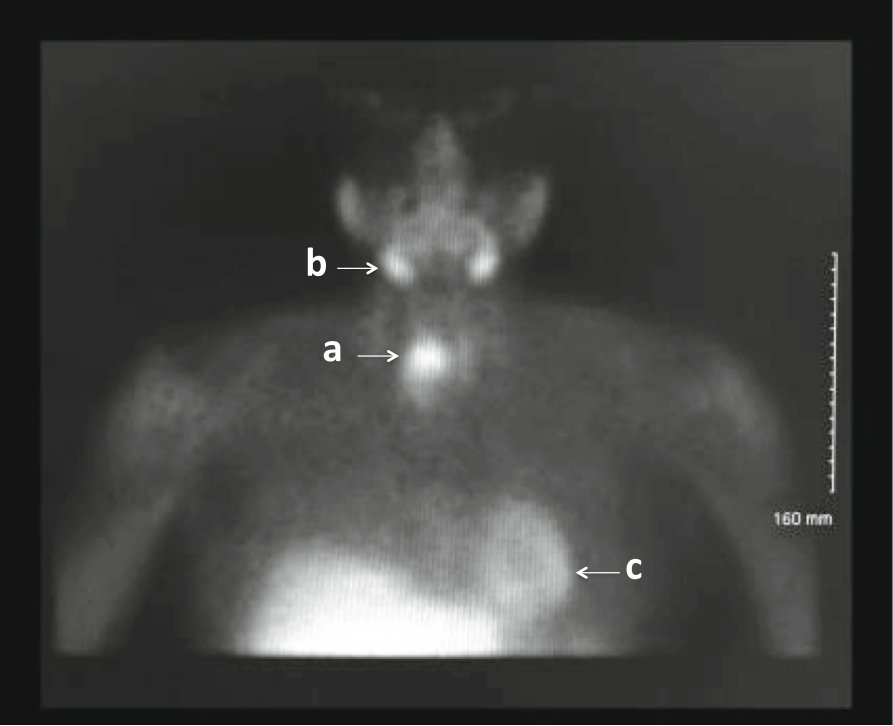
Technetium 99 m sestamibi scan showing intense tracer uptake relative to the right lower pole of thyroid (**a**), and physiological uptake in the salivary glands (**b**) and myocardium (**c**)

**Fig. 2:**
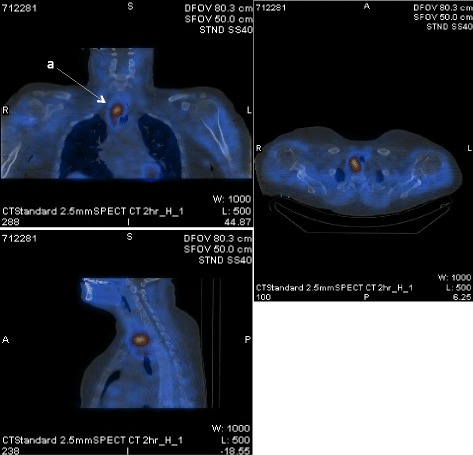
SPECT CT showing 3.7 cm soft tissue mass (**a**) in the right tracheoesophageal groove extending into the posterior upper mediastinum. There is some associated compression of the upper oesophagus which is slightly deviated to the left

Eight weeks prior to her ED visit, she was diagnosed with primary hyperparathyroidism (adjusted calcium 3.33 mmol/L (13.3 mg/dl) (normal range 2.2–2.6 mmol/L), PTH 367 (normal range 15–65) pg/ml) and 24 h urinary calcium 5.4 mmol/24 h (normal range 2.5–7.5 mmol/24 h). At her initial assessment she had no symptoms of hypercalcaemia apart from mild constipation. Family history was unremarkable. She received intravenous zolendronic acid and serum calcium fell to a nadir of 2.9 mmol/l. Neck ultrasound reported to show a 6 mm probable parathyroid lesion intimately related to the inferior pole of the right lobe of thyroid. One day prior to her acute presentation, and 6 weeks on from the ultrasound scan, a Sestamibi/SPECT CT revealed a 3.7 cm mass in the right tracheoesophageal groove extending to the posterior upper mediastinum at T1-T2 level (Figs. 1 & 2).

**Fig. 3:**
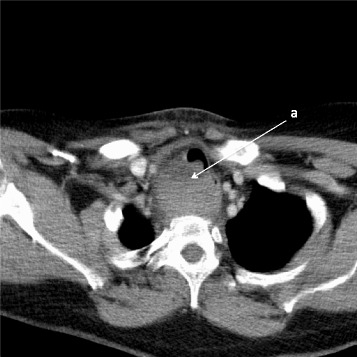
CT Neck showing large mass with a hypodense fluid level (**a**)

On arrival to ED, she was dyspnoeic and tachycardic. A biphasic stridor was noted. Flexible nasendoscopy identified no supraglottic or glottic cause for her stridor. The admitting medical team arranged a CT pulmonary angiogram to outrule a pulmonary embolism. CT thorax and subsequent emergency CT neck revealed significant increase in size of the mass since the scan 2 days prior, measuring 5.2 × 4.2 × 10.7 cm, with a hypodense fluid level, beginning at the level of the hypopharynx and extending into the superior mediastinum to the level of the aortic arch (Fig. 3). The trachea was narrowed with the smallest antero-posterior (AP) diameter measuring 0.4 cm at the level of the sternoclavicular joints.

**Fig. 4:**
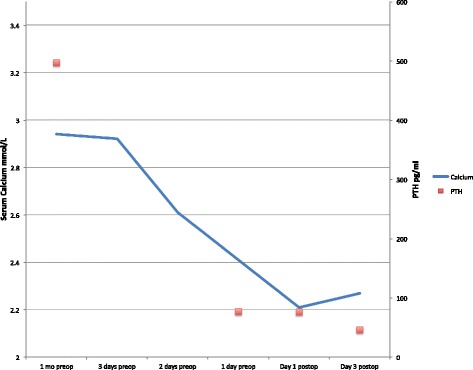
X20: H&E, objective lens X20. High power of parathyroid tissue showing bland nuclear features and lack of mitoses. There was no evidence of perineural or vascular invasion

**Fig. 5:**
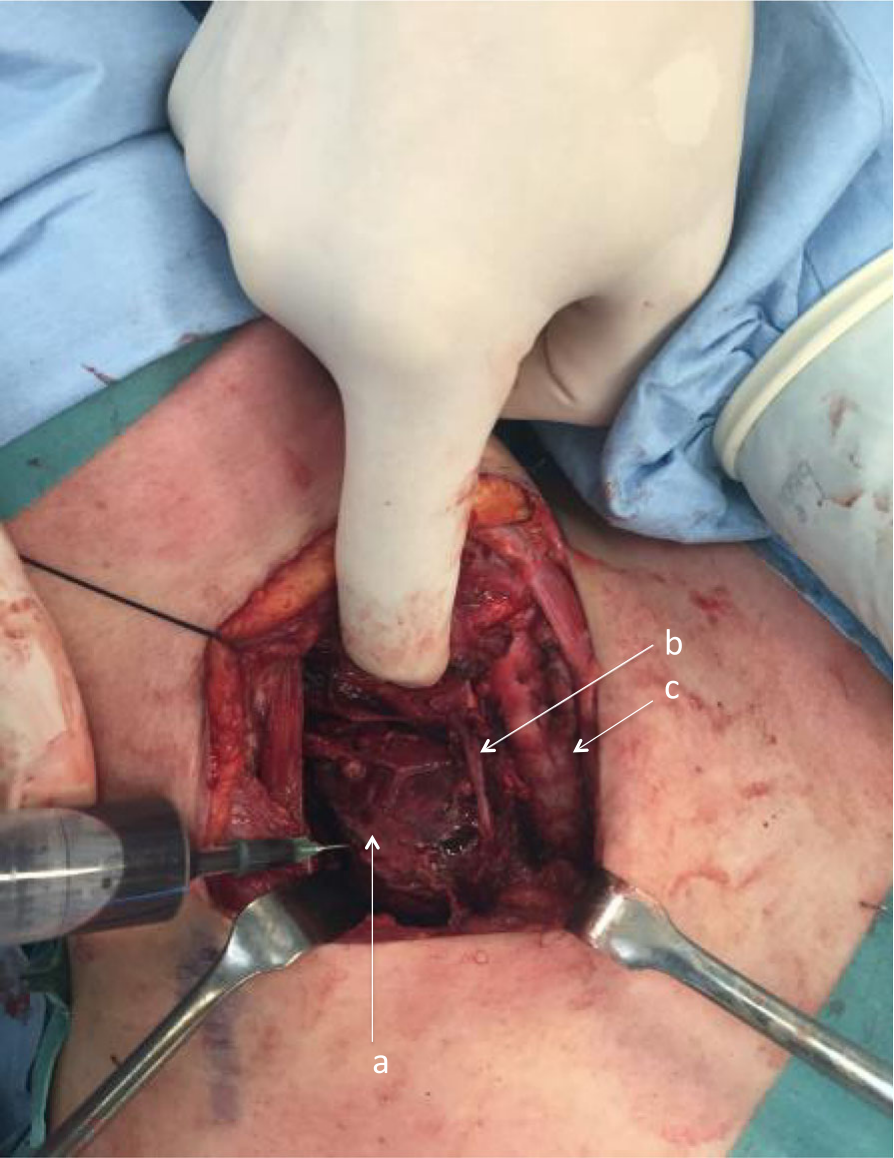
Trend in Calcium and PTH levels showing fall in PTH and calcium prior to excision of tumour

**Fig. 6:**
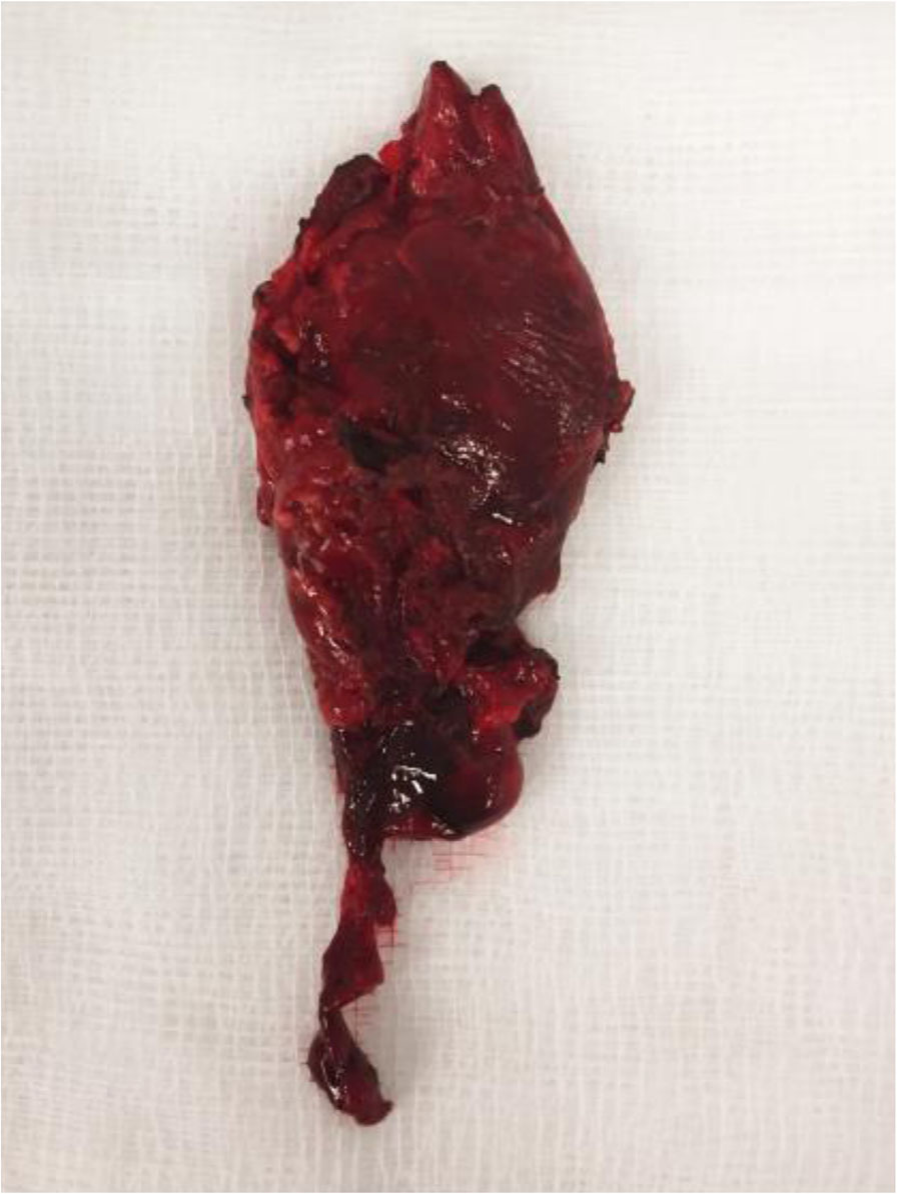
Aspiration of blood from parathyroid cyst prior to enbloc excision of the tumour. (**a**) parathyroid cyst, (**b**) recurrent laryngeal nerve, (**c**) tracheal rings

She was admitted to the intensive care unit, electively intubated and brought to theatre the following morning. Preoperatively calcium levels had normalised and PTH had fallen to 77 pg/ml from a peak value of 496 pg/ml suggesting infarction of the gland (Fig. 4). There was no tracheal invasion on bronchoscopy. The cystic mass was identified in level 6 posterior and inferior to the right hemi-thyroid with evidence of recent haemorrhage into the surrounding tissues. The right recurrent laryngeal nerve (RLN) lay over the tumour (Fig. 5). The close proximity of the tumour to the adjacent thyroid necessitated that a right hemi-thyroidectomy was performed with preservation of the RLN. Decompression of the cyst revealed old blood products and allowed complete dissection and removal of the tumour en-bloc (Fig. 6). Intra-operative PTH monitoring was not used.

**Fig. 7:**
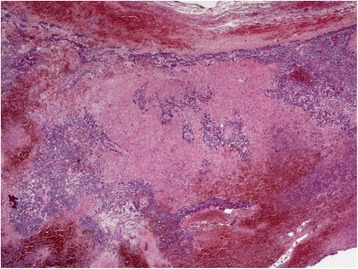
Gross specimen: Haemorrhagic parathyroid tumour

**Fig. 8:**
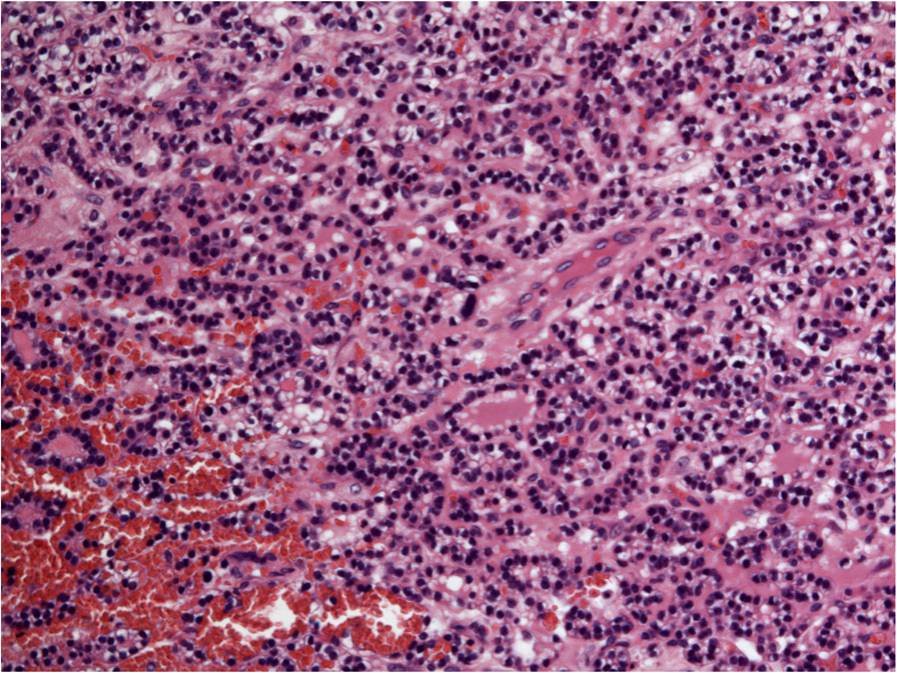
*X*2: H&E, objective lens *X*2. Periphery of gland showing marked haemorrhage within residual parathyroid tissue

She was extubated without complication on the first post-operative day. There was no stridor and vocal cord examination was normal. Histology confirmed a parathyroid neoplasm with extensive haemorrhage and necrosis; MIB-1 index <5% (Figs. 7 & 8). Serum calcium level remains normal 5 months post-operatively.

## Discussion

Spontaneous parathyroid haemorrhage is a rare but potentially life-threatening complication of parathyroid disease. *Capps* first reported a case of fatal haemorrhage due to rupture of a parathyroid adenoma in 1934. Since then, over 80 cases have been described in the English literature; ten of these associated with acute airway compromise requiring emergency intervention, and more than half of cases presenting with compressive symptoms due to haemorrhage.

Haemorrhage may occur into a parathyroid adenoma (cervical or ectopic), parathyroid hyperplasia or a parathyroid cyst and is thought to occur due to a lack of balance between cell growth and blood supply, somewhat similar to apoplexy seen in other endocrine neoplasia. It may be contained within the parathyroid gland, but often presents as extracapsular haemorrhage extending into the neck and/or mediastinum due to rupture of the haemorrhage through the relatively thin parathyroid tumour wall [1].

Clinical features are determined by the extent of the haemorrhage and degree of hyperparathyroidism. Spontaneous extracapsular parathyroid haemorrhage into the neck may present with acute painful neck swelling, discomfort and cervical ecchymosis [2]. External compression of the trachea can lead to airway compromise necessitating emergency intervention. Hoarseness and dysphagia may be presenting symptoms due to compression of the RLN and oesophagus respectively. Haemorrhage extending inferiorly into the mediastinum can manifest as cough, chest pain, dyspnoea, respiratory distress and may mimic aortic dissection. Haemorrhage has also been reported into hyperplastic parathyroid glandular tissue in secondary hyperparathyroidism in patients undergoing haemodialysis [3] and in one patient after suppression of PTH with cinacalcet [4]. The parathyroid gland may be functional or non-functional, and the serum calcium can range from high to low depending on the occurrence of gland infarction. Cases of parathyroid haemorrhage presenting as hypercalcemic crisis have been described [5]. Rarely, the underlying lesion may represent a parathyroid carcinoma [6].

Initial evaluation of an intracervical haematoma should include serum calcium levels; however patients may be normocalcemic. Imaging with CT, magnetic resonance imagine (MRI) and nuclear medicine is essential to delineate the anatomy of the haemorrhage and, if possible, identify the source of the haemorrhage [7]. In our case, although the ultrasound radiologist was experienced in neck ultrasound, it is likely that the adenoma was low down in the neck and was not seen on ultrasound hence the value of SPECT CT. The possibility of parathyroid haemorrhage should be considered in anyone presenting with retropharyngeal or mediastinal haemorrhage, particularly if there is a documented history of hyperparathyroidism. The use of anticoagulants should be avoided if possible, as the empiric administration of low molecular weight heparin in our case may have resulted in enlargement of the haematoma.

Surgical exploration is the treatment of choice and offers definitive management of the hyperparathyroidism aswell as addressing local haemorrhage; however timing remains controversial. Airway compromise necessitates emergency surgical exploration and evacuation of the haemorrhage, as in our case. Some suggest a watch-and-wait approach with surgery deferred to 3 months [8], particularly as many cases of primary hyperparathyroidism can be cured as a result of apoplexy of the gland [1]. Indeed, in our case the excised parathyroid gland was necrotic and the patient’s calcium had normalized before surgical intervention suggesting haemorrhagic infarction of the gland had led to autoparathyroidectomy. Recurrence of primary hyperparthyroidism following initial spontaneous remission has been described, suggestive that in some cases remission may be due to impaired perfusion of the tumour due to local pressure effects rather than gland necrosis[9], therefore careful biochemical monitoring is required.

## Conclusion

Spontaneous parathyroid haemorrhage is a rare but potentially life-threatening complication. The diagnosis should be considered in any patient presenting with a spontaneous cervical haemorrhage of unknown aetiology, particularly if there is evidence of hypercalcemia, history of hyperparathyroidism or ecchymosis of the neck or chest wall. Initial evaluation should include a calcium and PTH level although these may have fallen if significant glandular haemorrhage has led to infarction of the gland. While some propose conservative management, surgery is the preferred option, particularly in the presence of acute airway compromise.

## Abbreviations

AP: Antero-posterior
CT: Computed tomography
ED: Emergency department
MRI: Magnetic resonance imaging
PTH: Parathyroid hormone
RLN: Recurrent laryngeal nerve
SPECT: Single-photon emission computed tomography
