# Erratum to: Acute airway compromise due to parathyroid tumour apoplexy: an exceptionally rare and potentially life-threatening presentation

**DOI:** 10.1186/s12902-017-0193-3

**Published:** 2017-07-14

**Authors:** Aoife Garrahy, David Hogan, James Paul O’Neill, Amar Agha

**Affiliations:** 1 Department of Endocrinology, Beaumont Hospital, Dublin, Ireland; 2 Department of Otolaryngology, Head and Neck Surgery, Beaumont Hospital, Dublin, Ireland

## Erratum

The original article [1] was updated to correct the order of figure legends. Legend 4 has been moved to Fig. 8, and Legends 5, 6, 7 and 8 have been moved up to Figs. 4, 5, 6 and 7, respectively. This mistake was carried through by the production team handling this article, and thus was not the fault of the authors.
